# Real-Time Electrocardiogram Transmission from Mount Everest during Continued Ascent

**DOI:** 10.1371/journal.pone.0066579

**Published:** 2013-06-20

**Authors:** Wei-Fong Kao, Jyh-How Huang, Terry B. J. Kuo, Po-Lun Chang, Wen-Chen Chang, Kuo-Hung Chan, Wen-Hsiung Liu, Shih-Hao Wang, Tzu-Yao Su, Hsiu-chen Chiang, Jin-Jong Chen

**Affiliations:** 1 Department of Emergency and Critical Care Medicine, Taipei Medical University Hospital, Taipei, Taiwan; 2 Institute of BioMedical Informatics, National Yang-Ming University, Taipei, Taiwan; 3 Institute of Neuroscience, National Yang-Ming University, Taipei, Taiwan; 4 Institute of Physical Therapy, National Yang-Ming University, Taipei, Taiwan; 5 Department of Sports Information and Communication, National Taiwan College of Physical Education, Chiaryi, Taiwan; 6 Center for Measurement Standards, Industrial Technology Research Institute, Hsinchu, Taiwan; 7 Taiwan Telemedicine Device Company, Kaohsiung, Taiwan; 8 Yu-Shan National Park, Nan-Tou, Taiwan; King’s College London School of Medicine, United Kingdom

## Abstract

The feasibility of a real-time electrocardiogram (ECG) transmission via satellite phone from Mount Everest to determine a climber’s suitability for continued ascent was examined. Four Taiwanese climbers were enrolled in the 2009 Mount Everest summit program. Physiological measurements were taken at base camp (5300 m), camp 2 (6400 m), camp 3 (7100 m), and camp 4 (7950 m) 1 hour after arrival and following a 10 minute rest period. A total of 3 out of 4 climbers were able to summit Mount Everest successfully. Overall, ECG and global positioning system (GPS) coordinates of climbers were transmitted in real-time via satellite phone successfully from base camp, camp 2, camp 3, and camp 4. At each camp, Resting Heart Rate (RHR) was transmitted and recorded: base camp (54–113 bpm), camp 2 (94–130 bpm), camp 3 (98–115 bpm), and camp 4 (93–111 bpm). Real-time ECG and GPS coordinate transmission via satellite phone is feasible for climbers on Mount Everest. Real-time RHR data can be used to evaluate a climber’s physiological capacity to continue an ascent and to summit.

## Introduction

The rapid development of telecommunication technology has brought with it the potential for innovation to daily medical care. The physiological parameters of the heart can be evaluated using an electrocardiogram (ECG). ECG remains an important tool for the measurement of the overall function of the human heart. An ECG can be used to detect the warning signs of life threatening conditions including, hypoxia, ischemia, shock, and arrhythmia. In its early stages of use, heavy equipment and complicated cable wiring were required to record ECGs. With the advent and widespread use of advanced wireless technology, as well as, miniature ECG devices, mobile ECG recording devices have been developed and can be used for remote monitoring by physicians and other health care professionals.

Mount Everest is widely considered to be one of the most remote and harshest environments on our planet. As of May 21, 2009 there had been 4,102 ascents to the summit by about 2,700 individuals; however, 216 people have died during a summit attempt [1]. For each successful summit, the mortality rate is reported to be 5.3% when using supplemental oxygen; whereas, when summiting without the benefits of oxygen supplementation the mortality rate rises to 7.9%. It is common for a climber to be faced with the difficult decision upon reaching camp 3 and camp 4 on Mount Everest regarding whether a final ascent to the summit is possible. Currently, there are no objective guidelines to help guide climbers at this time, only monitoring of signs and symptoms of high altitude illness, which can be subjective and may not be accurately reported by each climber.

The feasibility of transmitting physiological data from the Mount Everest base camp, or telemedicine, was examined during the 1998 and 1999 Mount Everest climbing seasons. The Yale University School of Medicine cooperated with the National Aeronautics and Space Administration (NASA) to validate the feasibility of telemedicine. The project was considered to be an important cornerstone of telemedicine [2], [3].

To our knowledge, only one report published in 1975 details ECG data being transmitted from Mount Everest. At an altitude of 8848 meters a survey tripod was assembled at the summit in order to accomplish this feat for the first time [4]. Otherwise, no climber has transmitted real-time ECG data from above 7000 meters on Mount Everest [2], [3], [5]. We currently report the development of a satellite transmission system designed to transmit ECG and global positioning system (GPS) data from climbers during an ascent of Mount Everest.

## Methods

This prospective observational study was completed on Mount Everest, Nepal: April 13, 2009 (arrival at base camp); May 19, 2009 (travel from camp 4 to the summit and back to camp 4); and May 21, 2009 (return to base camp).This study was approved by the Institute Review Board of Taipei Veterans General Hospital. A consent form was signed by each climber prior to the study.

**Figure 1:**
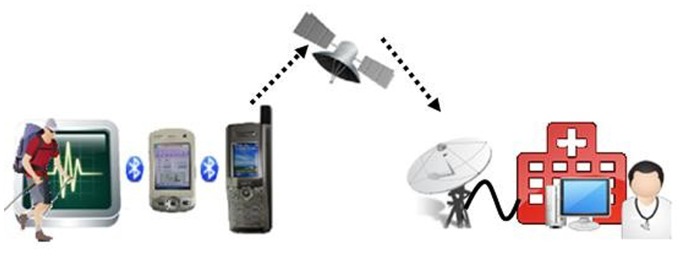
Illustration of the real-time ECG satellite transmission system. Left to Right: One lead ECG is transmitted via Bluetooth to a PDA, which forwards the signal to a satellite phone again through Bluetooth. The satellite phone acts as a modem and sends the data through a satellite to the servers in Taiwan, which then dispatches the ECG data to the smart phone and the PC of the team doctor.

Before the expedition, a remote health care team made up of physicians, engineers, and specialists in medical informatics, designed an ECG transmission system to transmit ECG data during the Everest ascent. The structure of the transmission system and the scale of each device are shown in Figure 1. The wearable one lead ECG sensor is based on Kuo’s design (TD1) and was modified for use with thick gloves commonly used during high altitude expeditions. Data acquisition and storage were similar to previously described procedures [6], [7]. Briefly, ECGs were recorded using a wireless sensor (5.2×3.1×1.2 cm, 11 g, KY202-BT, K&Y Lab, Taiwan). A precordial ECG was taken from each subject and the ECG signal was amplified 1000-fold and band-pass filtered (0.8 to 40 Hz). The ECG signal was then digitally acquired using an eight-bit analog-to-digital converter with a sampling rate of 250 Hz. The digitized ECG signals were analyzed on-line and wirelessly transmitted to a nearby PDA with the standard Bluetooth protocol.

The digital signal processing of the ECG was similar to the procedures used in previous studies [6], [7]. In the QRS identification procedure, the microcontroller first detected all peaks of the digitized ECG using a spike detection algorithm [8]. Parameters such as spike amplitude and duration were measured so that their means and standard deviations (SD) could be calculated as standard QRS templates. Each QRS complex was then identified, and each ventricular premature complex or noise was rejected according to its likelihood in the standard QRS template. The R point of each valid QRS complex was defined as the time point of each heart beat, and the interval between two R points (R-R interval) was estimated as the interval between current and latter R points. In the R-R interval rejection procedure, a temporary mean and SD of all R-R intervals was first calculated for standard reference. Each R-R interval was then validated: if the standard score of an R-R value exceeded 3, it was considered erroneous or non-stationary and was rejected. Finally, heart rate in beats-per-minute (bpm) was calculated by dividing 60 by the R-R interval.

Each member of the summit team was fitted with electrodes, and supplied with an ECG sensor with Bluetooth capability, a personal digital assistant (PDA) device, and a single satellite phone was shared by the summit team. The ECG device was wearable and light with a battery life of approximately 20 days. The three key components for the real-time ECG transmission system are shown in Figure 1C. The PDA was utilized because it was not possible to program the Thuraya phone running Windows CE [9] directly; however, this was overcome by using the Windows Mobile operating system. A Thuraya satellite phone was chosen since it has proven to be reliable on Mount Everest, has high bandwidth, and is light-weight. The total weight of the transmission system was 474.39 g (ECG sensor and connecting leads [45.54 g], PDA [140.79 g], satellite phone [140.12 g], and super sized battery [147.94 g]. The battery of the Thuraya satellite phone [10] was modified for a higher power capacity.

While the remote health care team received the ECG and GPS information in Taiwan, the climbers were also able to view the real-time data on their PDA. The team physician analyzed the data and made suggestions on whether individual climbers were suitable for further ascent. Maximum age-related heart rate (MAHR) was used to estimate each climber’s reserve capacity. MAHR is determined by the following equation:

According to the Centers for Disease Control (CDC), achieving a HR of 70–85% MAHR is representative of vigorous exercise. If the estimated HR at the subsequent altitude was greater than 85% MAHR, it was suggested that the climber not continue their ascent. Measurements were taken at base camp (5300 m), camp 2 (6400 m), camp 3 (7100 m), and camp 4 (7950 m) 1 hour after arrival, following a 10 minute rest period.

## Results

A total of 4 climbers (3 men; 1 woman) of Taiwanese descent with an average age of 39±6 (range: 34–48 years) were enrolled in the Mount Everest Summit program and ascended from the South side of the mountain (**Table 1**). Each climber had previously completed six of the Seven Summits, as well as Mount Cho Oyu (elevation of 8201 m), between 2006 and 2008. In 1995, climber #3 successfully reached the summit of Mount Everest from the North side.

**Table 1:** The baseline characteristics of 4 participants and the medication history in this climb.

Participant	Age	Gender	History of Climbing	Medication history
1	34	Male	Six of the Seven Summits and Mount Cho Oyu between 2006 and 2008.	None
2	35	Male	Six of the Seven Summits and Mount Cho Oyu between 2006 and 2008.	Diamox Serotide inhalor
3	38	Female	Summit Mount Everest from the north side in 1995 Six of the Seven Summitsand Mount Cho Oyu between 2006 and 2008.	None
4	48	Male	Six of the Seven Summits and Mount Cho Oyu between 2006 and 2008.	None

**Figure 2:**
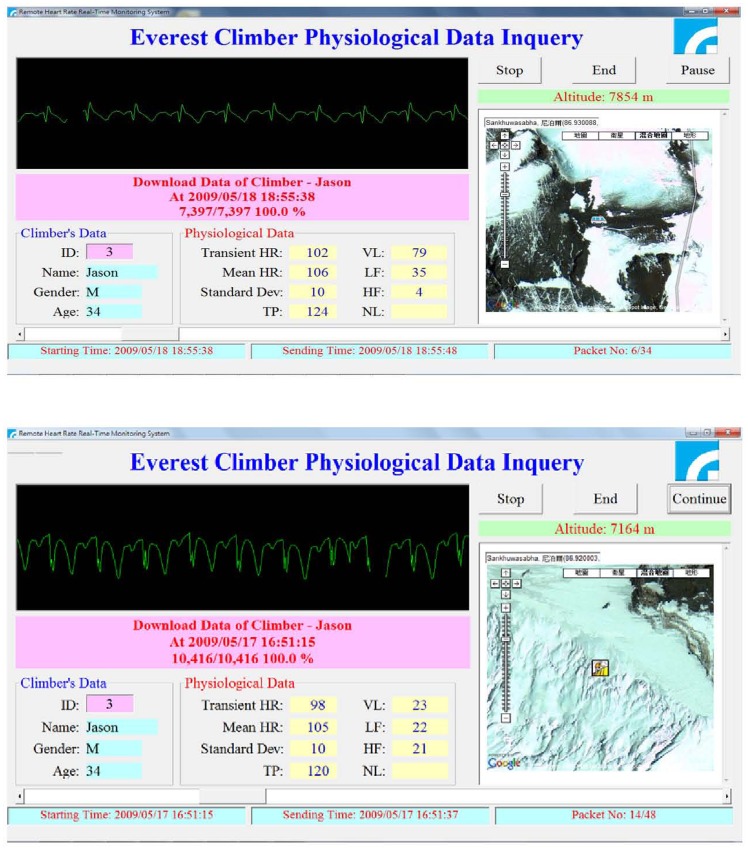
The screenshot of the ECG software interface at the hospital side. The upper right corner shows the elevation. The lower right corner shows his location on Google Earth. Top: At camp 4 (7854 m) the heart rate of the 34 year-old climber was 102 bpm. Bottom: At camp 3 (7164 m) the heart rate of the 34 year-old climber is 98 bpm.

The ECG of climbers was transmitted in real-time with GPS coordinates successfully from base camp, camp 2, camp 3, and camp 4, respectively, of Mount Everest to computers and mobile phones in Taiwan. The ECG of climber #1– a 34 year-old male – whose ECG was transmitted real-time via satellite phone from camp 3 on May 17^th^ and camp 4 on May 18^th^, 2009 is presented in **Figure 2**. The lowest RHR and MAHR of participants at each camp are presented in **Table 2**.

**Table 2:** The lowest resting heart rate of participants at each camp in Mount Everest.

	MAHR (bpm)#	Base camp¶	Camp 2¶	Camp 3¶	Camp 4¶
Participant		(5300 m; FiO_2_ = 21%)	(6400 m; FiO_2_ = 21%)	(7100 m; FiO_2_ = 23%; with O2 0.5 L/min)	(7950 m; FiO_2_ = 25%; with O2 1 L/min)
1	186	54 (29%)	112 (60%)	98 (53%)	93 (50%)
2	185	90 (49%)	115 (62%)	Withdraw	Withdraw
3	182	72 (40%)	112 (62%)	NM	NM
4	172	72 (42%)	94 94 (55%)	103 (60%)	NM

Withdraw: The particular participant didn’t reach this camp.

NM: The heart rate is not measured.

# Maximum age-related heart rate (MAHR) is calculated by maximal age minus related heart rate, and the unit is beat per minute (bpm).

¶ The environment characteristics for each camp, including the altitude (meter; m), fraction of inspired oxygen (FiO_2_), and the amount of mandatory oxygen supply, are shown.

The lowest resting heart rates of participants at each camp were presented as the value (%; the percentage compared to MAHR).

In total, 977 seconds (696 packets) of ECG signals were transmitted in real-time to Taiwan. Between them, 107 seconds of the signals were transmitted from base camp, 503 seconds from camp 2, 170 seconds from camp 3, and 197 seconds from camp 4. Clear ECG signals were seen in 580 packets (83.3%). Overall, the rate of artifact was 16.7%, with 9.8% at camp 4, 24.6% at camp 3, 13.7% at camp 2, and 30.4% at base camp.

The RHR transmitted from camp 4 was between 93 bpm and 111 bpm (50% and 59.7% of MAHR, respectively). From camp 3, RHR was between 98 and 115 bpm (52.6% and 66.9% of MAHR). The climbers started using oxygen with a flow rate of 0.5 l/min at camp 3. Climber #2– a 35-year old male climber whose RHR was between 113 and 130 bpm (61.1% and 70.3% of MAHR, respectively) at camp 2– discontinued his ascent due to symptoms of respiratory distress and eye pain. As a group, the RHR transmitted from camp 2 was between 94 and 128 bpm (54.6% and 68.8% of MAHR, respectively), and among the 3 participants who were able to summit, RHR from base camp was between 54 and 113 bpm (29% and 60.8% of MAHR, respectively).

## Discussion

The current study demonstrates real-time ECG and GPS coordinate transmission via satellite phone is feasible for climbers on Mount Everest. Using a modified, light-weight, wireless, transmission system, RHR for climbers on Mount Everest were sent to remote medical teams in order to evaluate each climber’s physiological capacity to continue an ascent and summit attempt. We achieved our goal by designing a system under 500 g that successfully transmitted climbers’ ECGs from each camp. An attempt at the top of Everest to transmit real-time ECG was made but failed due to satellite signal interference caused by thick clouds [11]. This is the first documented real-time ECG transmission from above 7000 m from Mount Everest using a light-weight satellite phone as the source of transmission. Similar attempts have been reported at lower altitudes, including from Camp 1 (5950 m) and Mount Aconcagua (6962 m) [11]. A transmission from 8848 m using a survey tripod that required construction by several Tibetan and Chinese climbers has also been reported [4].

Climbers on Mount Everest are at risk of developing potentially fatal high altitude illness (such as high altitude pulmonary edema [HAPE] and high altitude cerebral edema [HACE]) [12], [13]. Resting tachypnea and tachycardia are the major signs for HAPE [12]. Thus, beside the climbers’ hypoxic ventilatory response (HVR) and tachypnea on Mount Everest [14]–[16], resting heart rate may be another important indicator of physiological distress and is worth monitoring by each climber and their medical support group.

In order to adapt to the changing environment common at extremely high altitudes, the human body undergoes a process of acclimatization, including increased heart rate, respiratory rate, and hemoglobin. In the Yale/NASA E3 project (1998 and 1999) climbers transmitted their heart rate, temperature, respiratory rate, ECG, pulse oximetry, and accelerometer measurements through radio links back to base camp and the medical support group at Yale [2]. The MagIC project performed ECG recordings and real-time transmission at sea level and base camp via Bluetooth and WiFi [5]. In addition, ECGs were directly recorded to memory cards inserted into each climber’s vests at Everest camp 1 and camp 2. However, unlike the current study, no real-time transmission was attempted. Wagner had also transmitted one-lead ECG, respiratory rate, skin, and core temperature of a 41-year-old climber with an ECG sensor that weighed approximately 200 g from Mount Aconcagua (6962 m) [11]. A satellite phone was not necessary for the summit of Mount Aconcagua. The currently reported transmission system is different from the Yale/NASA and MagIC projects in that it is designed with the objective to be used at the top of Mount Everest. A defining characteristic of this transmission system is its low weight and overall durability. The primary objective was to provide a simple and useful reference of the climbers’ physical condition while at altitude and provide this information to a team leader and doctors at base camp.

As reliable technology becomes available to measure ECG and HR during ascent at high altitudes, estimated MAHR should be considered as another tool in the evaluation armamentarium during ascent. However, its exact role and importance in guiding decisions regarding further ascent will require further evaluation. None of the current subjects reached an MAHR greater than 62% at any point during ascent, including summit (Table 2). Subjects in Karliner et al reached 80.21% and 82.01% of their estimated MAHR at the summit in previous reports [17]. The upper limit of heart rate to be safely engaged in continuous vigorous physical activity, as defined by the CDC, is 85% of MAHR [18]. In Table 3, data derived from 2 previously published studies were compared with the estimated heart rate calculated by the currently reported methodology. There were no significant differences between measured and estimated values, respectively. Future studies evaluating criteria to predict safety during ascent should consider the results from the current study, although it is unclear what percentage of MAHR, when combined with other variables (ie, age, training, previous attempts, etc) will be best associated with a safe ascent.

**Table 3:** The differences between the measured heart rate (HR) published in the respective references and predicted heart rate (HR) calculated by method used in this study.

Study	Location	Altitude (m)	O_2_ supplement#	Mean measured HR	Predicted HR	Difference (%)
Ref. #18^a^	Base Camp	5400	Regular air	70		
	Camp 2	6300		80	82	2.1
Ref. #21^b^	Camp 2	6550	Regular air	77		
			1L O_2_	82		
			2L O_2_	80		
	Camp 4	8000	Regular air	100	94	−6
			1L O_2_	90	100	11.3
			2L O_2_	89	98	9.8

HR = heart rate; m = meter;

a The data were cited from the Reference #19. Predict HR at camp 2 = measured HR at base camp × 6300 m/5400 m; % of difference = percentage of the difference between measured HR and predict HR.

b The data were cited from the Reference #22. Predict HR at camp 4 = measured HR at camp 2 × 8000 m/6550 m.

# The heart rates under various conditions were measured, including without O_2_ supplement (regular air), 1L O_2_ supplement, or 2L O_2_ supplement.

Age is an important factor to consider for climbers considering a summit attempt. Dunin-Bell and Boyle [13] previously reported a 38-year-old male who suffered from high altitude pulmonary edema who presented with a heart rate of 125 bpm (68.7% of his MAHR) at base camp – much higher than the mean RHR of 70 bpm at base camp reported by Karliner et al [8], 83 bpm for follow-ups group at base camp reported by Wiseman et al [19], and 76 bpm by Agostoni et al [20]. Huey et al [21] reported that the likelihood of summiting Mount Everest declined dramatically after the age of 40 and mortality rate was increased dramatically after the age of 60. Age appears to be an important factor for safety and success during an ascent of Mount Everest.

In conclusion, an evaluation system that includes MAHR, age, and other variables requires further study in an effort to reduce the death rate not only on Everest, but all mountains at extreme altitudes. Utilizing current and evolving technology to measure and transmit ECG data, will allow climbers and the medical support group to determine real-time heart rate reserve. The use of a real-time ECG transmission system may protect climbers from risky summit attempts, but could also increase the database of climber data from camp 3 and camp 4.

### Limitations

Some limitations of our study must be taken into account, including a small number of participants. The ECG signals were not transmitted real-time by all the climbers and not from the summit of Mount Everest. Furthermore, the satellite phone was shared, therefore climbers needed to transmit ECG data separately which delayed transmission of some data. The equation has not been verified using well controlled studies. Taken together, additional studies should be conducted to address these limitations.

## References

[1] Anonymity (2009) http://www.8000ers.com/cms/content/view/52/185/.Accessed 5 May 2011.

[2] Angood PB, Satava R, Doarn C, Merrell R, E3 Group (2000) Telemedicine at the top of the world: the 1998 and 1999 Everest extreme expeditions. Telemed J E Health 6: 315–325. 11110635 10.1089/153056200750040174

[3] Satava R, Angood PB, Macedonia C, Merrell R (2000) The physiologic cipher at altitude: telemedicine and real-time monitoring of climbers on Mount Everest. Telemed J E Health 6: 203–313. 10.1089/153056200750040165 11110634

[4] Wu T, Li S, Ward MP (2005) Tibetans at extreme altitude. Wilderness Environ Med 16: 47–54. 15813148 10.1580/pr04-04.1

[5] Di Rienzo M, Meriggi P, Rizzo F, Astiglioni P, Lombardi C, et al (2010) Textile technology for the vital signs monitoring in telemedicine and extreme environments. IEEE Trans Inf Technol Biomed 14: 711–717. 20421189 10.1109/TITB.2010.2048921

[6] Kuo TB, Lin T, Yang CC, Li CL, Chen CF, et al (1999) Effect of aging on gender differences in neural control of heart rate. Am J Physiol 277: H2233–H2239. 10600841 10.1152/ajpheart.1999.277.6.H2233

[7] Liu CC, Kuo TB, Yang CC (2003) Effects of estrogen on gender-related autonomic differences in humans. Am J Physiol Heart Circ Physiol 285: H2188–H2193. 12881217 10.1152/ajpheart.00256.2003

[8] Kuo TB, Chan SH (1992) Extraction, discrimination and analysis of single-neuron signals by a personal-computer-based algorithm. Biol Signals 1: 282–292. 1307929 10.1159/000109333

[9] Anonymity (2011) Windows CE 2011. http://msdn.microsoft.com/en-us/library/ms905511.aspx. Accessed 5 May 2011.

[10] Thuraya satellite phone (2011) http://www.thuraya.com/.Accessed 5 May 2011.

[11] Grocott MP, Martin DS, Levett DZ, McMorrow R, Windsor J, et al (2009) Arterial blood gases and oxygen content in climbers on Mount Everest. N Engl J Med 360: 140–149. 19129527 10.1056/NEJMoa0801581

[12] Litch JA, Bishop RA (2001) Reascent following resolution of high altitude pulmonary edema (HAPE). High Alt Med Biol 2: 53–55. 11252699 10.1089/152702901750067927

[13] Dunin-Bell O, Boyle S (2009) Secondary prevention of HAPE in a Mount Everest summiteer. High Alt Med Biol 10: 293–296. 19775220 10.1089/ham.2008.1094

[14] West JB (1982) American Medical Research Expedition, to Everest, 1981. Physiologist 25: 36–38. 7079304

[15] West JB (1983) American Medical Research Expedition to Everest: a study of man during extreme hypoxia. Prog ClinBiol Res 136: 431–441. 6665025

[16] West JB (1984) Human physiology at extreme altitudes on Mount Everest. Science 223: 784–788. 6364351 10.1126/science.6364351

[17] Karliner JS, Sarnquist FF, Graber DJ, Peters RM, West JB (1985) The electrocardiogram at extreme altitude: experience on Mt. Everest. Am Heart J 109: 505–513. 3976477 10.1016/0002-8703(85)90555-1

[18] National Center for Chronic Disease Prevention and Health Promotion (2011) Target Heart Rate and Estimated Maximum Heart Rate. 2011. Available: http://www.cdc.gov/physicalactivity/everyone/measuring/heartrate.html. Accessed 5 May 2011.

[19] Wiseman C, Freer L, Hung E (2006) Physical and medical characteristics of successful and unsuccessful summiteers of Mount Everest in 2003. Wilderness Environ Med 17: 103–108. 16805146 10.1580/pr45-04.1

[20] Agostoni P, Caldara G, Bussotti M, Revera M, Valentini M, et al (2010) Continuous positive airway pressure increases haemoglobin O2 saturation after acute but not prolonged altitude exposure. Eur Heart J 31: 457–463. 19903683 10.1093/eurheartj/ehp472

[21] Huey RB, Salisbury R, Wang JL, Mao M (2007) Effects of age and gender on success and death of mountaineers on Mount Everest. Biol Lett 3: 498–500. 17698450 10.1098/rsbl.2007.0317 PMC2391200

